# The perception of safety regarding the transfer of infants from the neonatal intensive care unit to a level II neonatology department: a mixed-method cohort study using a Safety-II approach

**DOI:** 10.1186/s12887-025-05537-4

**Published:** 2025-03-17

**Authors:** Karen de Bijl-Marcus, Fenna Mossel, Kees Ahaus, Bettine Pluut, Manon Benders, Arjan Bruintjes, Martina Buljac-Samardzic

**Affiliations:** 1 Department Neonatology, Wilhelmina Children’s Hospital, University Medical Center Utrecht, Utrecht University, Utrecht, The Netherlands; 2 Pluut & Partners, Vianen, The Netherlands; 3 Department Health Services Management & Organisation Erasmus School of Health Policy & Management, Erasmus University Rotterdam, Rotterdam, The Netherlands; 4 Regional Ambulance Service Utrecht (RAVU), Utrecht, The Netherlands

**Keywords:** Safety, Transfer, Safety-II, Neonatal intensive care, Safety perception

## Abstract

**Objective:**

This study aimed to investigate the perceived safety during the transfer process of infants from a Neonatal Intensive Care Unit (NICU) to a regional level II department. It sought to identify stakeholder agreements and divergences on safety and to determine the facilitators and barriers to achieving a high level of perceived safety.

**Design:**

This study employed a mixed-method cohort design and action research approach grounded in Safety-II principles.

**Setting:**

The study focused on transfers from a single Dutch university hospital NICU to multiple regional level II neonatology departments.

**Methods:**

Surveys were administered to parents and care professionals, including NICU staff, level II department staff, and ambulance personnel. The surveys consisted of both quantitative and open-ended questions. Data were analysed quantitatively and qualitatively, incorporating Safety-I and Safety-II perspectives, to assess the perceived safety and identify facilitators and barriers.

**Results:**

A total of 46 transfers were evaluated by 239 stakeholders. The overall perception of safety was positive among all stakeholder groups. There were no significant differences in the overall level of perceived safety between parents and care professionals. However, stakeholder perceptions varied significantly across transfer phases. Qualitative analysis revealed facilitators and barriers related to timing, parental participation and information exchange.

**Conclusion:**

This study indicated consistently positive safety perceptions among parents and care professionals. Effective communication, parental participation and optimal timing were identified as crucial for enhancing safety perceptions during transfers.

**Supplementary Information:**

The online version contains supplementary material available at 10.1186/s12887-025-05537-4.

## Introduction

The prevalence of preterm birth is increasing, with approximately 15 million preterm infants born worldwide each year. Preterm birth is the leading cause of death among children under the age of five years [1, 2]. To improve short- and long-term outcomes for high-risk infants, many countries have implemented regionalized and specialized neonatal care systems [3, 4]. However, this has resulted in neonatal intensive care units (NICUs) operating at full capacity, driven by improved survival rates, the treatment of increasingly complex and immature infants, and staffing challenges [5–11]. As a result, in the Netherlands infants who no longer require highly specialized care are often transferred to regional level II neonatology departments to ensure optimal utilization of NICU resources and access to appropriate levels of care (see Additional file 1 for neonatal care levels) [12–16]. Furthermore, transferring stable infants to a regional level II hospital brings them closer to home, allowing parents and local care professionals to become acquainted with each other prior to discharge.

However, transferring infants from a NICU to a level II neonatology department is not without risks. In 38% of cases, infants experience a transient physical decline following interhospital transfer [17]. Moreover, the transfer adds complexity to the care process for these still-vulnerable, recovering infants and may disrupt continuity of care, potentially affecting (the perceived) patient safety and satisfaction [18]. Previous studies have primarily focused on *parental perceptions of the safety* of the transfer process. Their findings indicate that parents often experience distress related to interhospital transfer, identifying three major sources of concern: (1) a lack of communication between NICU staff and parents, (2) the transfer process itself, and (3) variations in care practices during and shortly after transfer [19–32].

Limited research exists on how *care professionals* perceive the safety of the transfer process and how their views align with parental perceptions. Only two studies have examined care professionals’ perspectives on the perceived safety and quality of care during the transfer process. Hanrahan et al. conducted focus group meetings with parents, physicians, nurse practitioners, and nurses from the NICU and level II departments to explore their experiences with the transfer process [25]. Many issues raised by participants in this study focused on promoting optimal communication [33, 34]. Helder et al. conducted a qualitative explorative study by interviewing parents and nurses from NICU and level II neonatology departments [32]. Their interviews explored parents’ positive and negative experiences during the transfer process and how nurses recognized these experiences. *Trust* emerged as a central theme for parents in their study: gaining trust, betrayal of trust, and rebuilding confidence following the transfer. Both studies provided valuable insights into parents’ perceived safety and the role of care professionals in enhancing these experiences. However, little is known about how care professionals perceive the safety of transferring vulnerable infants to a lower level of care in a high-workload environment and how their views align with parental experiences. Studies in other patient groups have shown differences in safety perceptions between patients/families and care professionals, as well as variation among care professionals [35–39]. Exploring the safety perspectives of all involved care professionals, beyond just hospital physicians and nurses, could yield valuable insights.

In recent years, there has been a notable shift in the field of safety evaluation from the traditional “Safety-I” approach to the emerging *“Safety-II”* approach [40]. Safety-I primarily focuses on preventing failures and incidents by identifying and eliminating risks. It follows a reactive and retrospective model, emphasizing compliance with safety regulations and procedures. On the other hand, Safety-II recognizes that organizations and systems are complex and adaptive. It acknowledges that incidents and failures are not the sole indicators of safety and that studying *successful* outcomes and *normal operations* also provides valuable insights. It aims to understand how systems function effectively under normal circumstances. Safety-II considers the realities of the workplace (e.g., complexity, dynamism, numerous interactions, and human factors), requiring flexibility and resilience in work processes and among stakeholders to reinforce what goes right and prevent adverse outcomes [40, 41]. Evaluating and learning from routine transfers of infants from the perspectives of all relevant stakeholders, focusing on how these complex processes typically succeed, aligns well with the Safety-II principles.

This study aimed to:

(1) To investigate stakeholders’ perceptions of safety during the transfer process, including parents, physicians, physician assistants (PAs), nurses, administrative staff, and ambulance personnel. We will identify areas of agreement and divergence among stakeholder perspectives.

(2) To identify facilitators and barriers to achieving a high level of perceived safety during the transfer process. A detailed analysis of transfers perceived as *safe* will offer valuable insights into enhancing safety.

## Methods

### Study design

This mixed method cohort study presents the diagnostic phase of an action research project that aimed at the understanding and improving the perception of safety during the transfer process. Action research is a research approach in which transformative change is pursued through the simultaneous process of taking action and gaining knowledge through critical reflection with all participants. The participative, reflective nature of action research aligns well with the complexity of the transfer process, where multiple stakeholders are involved (aim 1) and where a Safety-II perspective can promote a safe transfer process (aim 2) [42–46]. Combining closed and open questions in a survey allowed us to address both our research aims. The quantitative component measures the safety perceptions of multiple stakeholders while identifying areas of (dis)agreement (first aim). The combination of closed and open questions explores the facilitators and barriers to achieving a high level of perceived safety (second aim). Given that these facilitators and barriers have not been fully identified in previous studies, a qualitative component is essential in addition to the quantitative component.

### Period

The transfer evaluations were conducted between 1 January 2022 and 1 June 2022, reflecting the period prior to the implementation of improvement actions.

### Setting

The present study focused on the transfer of infants from a NICU department to a regional level II department. The NICU of the Wilhelmina Children’s Hospital is part of a Dutch university hospital with 24 level NICU beds and a full range of pediatric medical subspecialists. The NICU is situated in a highly regionalized area with four regional post-NICU/High care (HC) departments and three non-HC units. A high percentage (> 90%) of infants are transferred to regional level II hospitals for convalescent care. Five regional level II neonatology departments participated in this study. Transportation occurred by ambulance. Parents were encouraged to familiarize with the receiving hospital through a physical/online tour prior to the transfer.

### Participants

All consecutive infants who were transferred from the NICU to a regional level II neonatology department were eligible for inclusion in our study when at least one of the parents could speak Dutch or English. There were no other inclusion or exclusion criteria. Care professionals involved in the transfer process of included infants were asked consent to participate in the study. This included:

- Nurses, physicians, PAs, and administrative staff working at the NICU.
- Nurses, physicians, PAs/nurse practitioners working in level II departments of hospitals participating in the present study.
- Ambulance personnel that conducted the transfer of the infant.

### Surveys

For each enrolled infant that was transferred, we invited the aforementioned care professionals (see: ‘participants’) to complete an online survey to describe their experiences with the transfer process of this specific infant. Each transfer was given a unique identification number. The survey’s content validity was established through interviews with parents (*n* = 3) and care professionals (*n* = 15, including all stakeholders), and by reviewing the pre-existing literature to ensure that all aspects of the transfer experience for parents and care professionals were considered. The survey consisted of quantitative (Likert scale or grading) and open-ended written questions (see Additional file 2).

NICU professionals, administrative staff, and ambulance personnel completed a written or online survey immediately after the transfer of the infant from the NICU to the level II neonatology department. Level II care professionals completed an online survey one week after the transfer. They gained access to the online survey directly after the transfer to prevent recall bias. Parents completed a survey via an interview setting by telephone approximately one week after the transfer, conducted by a research nurse not involved in the care process and not involved in the data analysis process.

### Quantitative analysis (1st & 2nd aim)

Quantitative data regarding the perception of safety were analysed using SPSS version 26. Significant differences between medians (Mann-Whitney U test) and mean ranks (Kruskal-Wallis test) was set at *p* < 0.05. Parents were not included as a stakeholder group in all comparative analyses due to differences in response category for some questions: a Likert scale for care professionals versus a dummy response category for parents.

### Qualitative analysis (2nd aim)

Constant comparative qualitative methods were used to analyse open-ended questions. Content analysis was performed by four investigators independently for identifying the thematic content. The investigators then agreed on overarching themes regarding facilitators and barriers to a safe transfer perception using a method comparable to the “Colaizzi’s descriptive phenomenological method” described by Morrow et al. [47]. This descriptive phenomenological method is a step-by-step approach in which texts are read and re-read, themes are coded and after clustering of these themes, the investigated phenomena ‘light up’. These phenomena are then articulated in simple terms and validated by participants - in this case, through focus groups. Five focus group meetings were conducted in which participated: parents (*n* = 3), representatives of infant association (*n* = 2), ambulance personnel (*n* = 1), physicians NICU (*n* = 4), nurses NICU (*n* = 4), physicians level II department (*n* = 6), nurses level II department (*n* = 7), administrative staff (*n* = 2), (action)researchers (*n* = 5). The focus groups were intended to present and interpret the collected results. This led to a deeper understanding of the factors that play a role in the experience of the participants.

### Data

The datasets used and analysed during the current study are available from the corresponding author on reasonable request.

### Ethics approval

The study was exempt from formal approval under the Dutch Medical Research Involving Human Subjects Act, as determined by the Medical Research Ethics Committee of the Erasmus School of Health Policy & Management, Erasmus University Rotterdam (ETH2021-0098) and of the Medical Research Ethics Committee of the University Medical Centre Utrecht. Additionally, the Ethical Review Committee of the University of Humanistic Studies (2022-03) conducted a substantive integrity assessment of the research plan. Participants provided written informed consent, and data were analysed anonymously. We did not encounter any ethical issues during the study.

## Results

During the study period, 70 infants met the inclusion criteria. Of these, 19 were transferred before parents were informed and/or had given their consent for inclusion in the study. Five parents declined participation. The remaining 46 infants were included in the study. These 46 transfers were evaluated by 239 stakeholders (see Table 1). Although all stakeholders were invited to participate in each eligible transfer, the response rate varied between two and ten surveys per individual transfers.

**Table 1:** Descriptive sample

Stakeholder	*n*	%	Survey topic		
			Preparation	Transfer	Admission Level II
NICU: Physician/PA	44	18	✓	✓	NA
NICU: Nurse	44	18	✓	✓	NA
NICU: administrative staff	45	19	✓	NA	NA
Ambulance personnel	31	13	✓	✓	NA
Level II: Physician/PA	16	7	✓	✓	✓
Level II: Nurse	20	9	✓	✓	✓
Parents	39	16	✓	✓	✓
**Total**	**239**		**231 of 239**	**173 of 194**	**63 of 75**

NA = not applicable; PA = physician assistant

### Perceived safety regarding the transfer process by stakeholders: *1st aim*

#### Perception of safety: similarities and differences

Table 2 presents the overall perception of safety reported by various stakeholders. Median scores indicated a *consistently positive perception* of the overall safety of the transfer process among parents and healthcare professionals. All parents perceived the overall transfer process as safe. There were *no statistically significant differences* between stakeholder groups (Kruskal-Wallis *H* (4) = 1.02, *p* > 0.05).

Table 3 shows the degree of perceived safety among different stakeholders for three key phases of the transfer process. Overall, *perceived safety remained high across all stakeholder groups in all three phases*, with median scores ranging from 8 to 9 The Kruskal-Wallis test revealed *significant differences between stakeholder groups* regarding perceived safety during the transfer phase (Kruskal-Wallis *H* (5) = 12.39, *p* = 0.03) and the admission phase (Kruskal-Wallis *H* (2) = 9.24, *p* = 0.01), but not during the preparation phase (Kruskal-Wallis *H* (6) = 9.02, *p* = 0.17). Pairwise comparison indicated that parents perceived significantly higher safety during the transfer compared to healthcare professionals working in level II or NICU departments. Similarly, parents reported a significantly higher perception of safety during admission to the level II department than healthcare professionals working in that department. Additionally, ambulance personnel perceived the safety of the transfer significantly higher than healthcare professionals in the level II departments.

**Table 2:** Evaluation of perceived overall safety (scale 1–5) categorised into different stakeholder groups

	Stakeholder	*N*	Median	1 Strongly disagree %	2 Disagree %	3 Neutral %	4 Agree %	5 Strongly agree %	*N*.O.
**Overall safety of** **the transfer**	Physician/PA NICU	44	4	5	0	0	50	36	9
	Nurse NICU	41	4	2	2	2	39	44	10
	Ambulance personnel	30	5	13	3	0	23	60	0
	Physician/PA (II)	16	4	0	0	0	56	44	0
	Nurse (II)	20	4	0	0	0	55	45	0

N.O. = no opinion; PA = physician assistant; II: Care professional from a level II department

* Significant differences between mean ranks *p* < 0.05 (Kruskal-Wallis test)

**Table 3:** Degree of perceived safety by the different stakeholders for the three phases in the transfer process: preparation, the actual transfer, and admission phase

Perceived safety (grade) during 3 transfer phases \Stakeholder		NICU			Ambulance personnel	Level II department		Parents
		Physician/ PA	Nurse	Administrative staff		Physician/ PA	Nurse	
**1. Preparation of transfer**	Median	8	8	8	8	8	8	8
	Mean Rank	106.99	112.70	134.99	126.35	92.28	101.30	177.37
	*N* = 231	43	43	44	31	16	20	34
2. Transfer *	Median	8	8	NA	8	8	8	9
	Mean Rank	80.81	81.63	NA	101.52	72.94	73.08	107.94
	*N* = 173	43	38	NA	31	16	20	25
3. Admission level II hospital*	Median	NA	NA	NA	NA	8.0	8.0	8.5
	Mean Rank	NA	NA	NA	NA	27.97	25.80	38.98
	*N* = 63	NA	NA	NA	NA	16	20	27

Grade 1–10: 1 least optimal, 10 most optimal; NA = not applicable; PA = physician assistant

* Significant differences between groups are based on mean ranks *p* < 0.05 (Kruskal-Wallis test)

### Facilitators and barriers to a high degree of perceived safety: ***2nd aim***

To investigate which aspects facilitate or hamper the level of perceived safety, stakeholder groups were asked several questions (see TABLE 4AB).

**Table 4:** AB. Evaluation of the timing of the transfer & information exchange (grade 1–5)

Table 4A: TIMING OF TRANSFER	Stakeholder	*N*	Median	Mean rank	1 Strongly disagree %	2 Disagree %	3 Neutral %	4 Agree %	5 Strongly agree %	*N*.O.
**Optimal timing transfer**	Physician/PA NICU	44	4	74.93	0	9	16	43	23	9
	Nurse NICU	44	4	70.86	14	11	14	23	36	2
	Ambulance personnel	30	4.5	84.83	0	10	10	13	33	33
	Physician/PA (II)	16	4	49.84	6	38	0	56	0	0
	Nurse (II)	20	4	60.05	0	25	10	60	5	0

Table 4B: **INFORMATION EXCHANGE**	**Stakeholder**	**N**	**Median**	**Mean rank**	**1** **Strongly disagree** **%**	**2** **Disagree** **%**	**3** **Neutral** **%**	**4** **Agree** **%**	**5** **Strongly agree** **%**	
**Receiving** sufficient & timely information from the NICU professionals	Administrative staff NICU	43	4	59.69	5	9	0	67	19	
	Ambulance personnel	31	4	52.05	3	19	7	55	16	
	Physician/PA (II)	16	4	50.06	0	13	6	81	0	
	Nurse (II)	20	4	56.20	0	10	5	75	10	
	Parents	38	NA	NA	No %	Yes %				
					24%	76%				
**Providing** sufficient & timely information to level II	Physician/PA NICU	44	4	40.48	0	7	9	59	25	
	Nurse NICU	43	4	47.60	5	5	2	46	42	
**Providing** sufficient & timely information to parents	Physician/PA NICU	43	4	44.28	0	5	14	60	21	
	Nurse NICU	43	4	42.72	5	12	12	44	28	

PA = physician assistant; N.O. = no opinion;

II: Care professional from a level II department

* Significant differences between mean ranks *p* < 0.05 (Kruskal-Wallis test)

#### Timing of the transfer

Table 4A presents the perception of the timing of the transfer as one of the facilitators/barriers. The Kruskal-Wallis test revealed no significant differences between stakeholder groups overall (Kruskal-Wallis *H* (4) = 8.83, *p* = 0.066). Pairwise comparison showed that the perception of ambulance personnel regarding the timing of the transfer was significantly more positive compared to the perspective of NICU professionals (*p* < 0.05).

In addition, respondents were asked to select a reason why the timing of transfer was not optimal. Of the respondents, 63% claimed that another time during the day would have been better, and 9% believed that the preparation for transfer was not complete. None of the respondents believed that the timing was inadequate based on the patient’s care needs. The remaining respondents did not select any of the predefined categories. Level II care professionals perceived the transfer as safer when it occurred during the day shift. When patients were transferred during the late afternoon/evening, level II care professionals felt that they could not provide the best possible care, as fewer staff were working in the department, which negatively influenced their perception of safety. In one quarter of cases (26%), parents evaluated the timing of the transfer of their infants as suboptimal, often related to uncertainties and lack of information regarding the exact timing. Preparing parents for these uncertainties and explaining the reasons behind them helped facilitate the process and enhanced trust.

#### Information exchange

The exchange of information was evaluated as one of the facilitators/barriers (see Table 4B). The Kruskal-Wallis test revealed no overall significant differences between stakeholder groups regarding receiving information. Regarding providing information, Mann-Whitney U test showed no differences between physicians/PAs and nurses (NICU). In addition, comparing the evaluation of receiving and giving information among stakeholder groups showed overall significant differences (Kruskal-Wallis *H* (5) = 12.52, *p* = 0.03). Pairwise comparisons showed that NICU nurses were more positive than physicians/PAs and nurses from the receiving hospital, but also than administrative staff and ambulance personnel. These findings indicate that care professionals receiving information were less satisfied with the information exchange than those providing it. Parents indicated that they were sufficiently and promptly informed by NICU staff in 76% of cases. In addition, in parents’ perception level II care professionals were well informed by the NICU professionals in 84% of cases. This is in line with the perception of NICU professionals.

The qualitative analyses indicate that discrepancies between (the interpretation of) discharge criteria or type of care that can be delivered in specific level II departments increased the feeling of unsafety perceived by parents and/or level II care professionals. Both NICU and level II care professionals, as well as parents, expressed the need for clearer and more uniform discharge criteria that are consistently shared with parents. Parents appreciated receiving repeated information, starting soon after NICU admission, preferably using multiple modalities (written, oral, and visual information). Personal contact between parents and level II / ambulance care professionals prior to the transfer facilitated the safety perception of parents. Furthermore, involving parents in the exchange of information between parties (NICU, ambulance, and level II) fostered parental empowerment and a sense of control. For example, in some cases, parents were asked to document specific aspects that care professionals should be aware of from their perspective. This written parental handover was subsequently given to the level II care professionals, facilitating them to deliver optimal care and meeting the expectations of parents. In complex cases, the level II care professionals received a discharge letter and radiological images prior to the transfer of the infant in addition to a handover conversation in which parents, referring and receiving care professionals participated through an online meeting. These examples of Safety-II behaviour were highly valued by both parents and level II care professionals and reinforced the perception of safety.

#### Concerns

The Kruskal-Wallis test revealed no overall significant differences between stakeholder groups regarding having concerns during the transfer process. Pairwise comparisons also did not reveal any differences between groups. Prior to the transfer, 20–25% of the care professionals working at the NICU had concerns regarding the transfer. One-third of the parents experienced concerns regarding the transfer. These concerns were mainly related to fear of the unknown and worries about their infant’s comfort during the transfer process. Most parents had a great deal of confidence in the knowledge, skills, and expertise of the healthcare providers. When a familiar care professional accompanied their infant during the transfer, or when parents could accompany their infant themselves, this enhanced their perception of safety. Most parents felt reluctant to share their concerns with care professionals, believing their worries were “normal” and not wanting to “burden care professionals with them”. When care professionals actively asked parents about their concerns, it helped to reduce their fears and uncertainties. When a care professional, despite a high workload, exuded calmness and took the time to provide personal attention to parents and their thoughts, it fostered trust. Table 5 summarises the facilitators and barriers to a high degree of perceived safety.

**Table 5:** Summary of facilitators and barriers to a high Degree of perceived safety

	FACILITATOR
**TIMING OF TRANSFER**	Transfer in the morning/early afternoon
	Complete preparation
	Preparing parents for the uncertainties regarding the exact timing of the transfer and explaining the reasons behind those uncertainties
**INFORMATION EXCHANGE**	Adhering to uniform discharge criteria
	Sharing discharge criteria with parents soon after admission to the NICU
	Understanding which types of care can and cannot be provided by specific level II facilities
	Informing parents soon after admission to the NICU regarding the transfer
	Providing parents with transparent, consistent, and repeated information using multiple formats (oral, written, visual)
	Knowing which information, the receiving care professionals need to deliver optimal care
	Sharing written handovers and diagnostic images prior to the transfer with care professionals of the level II department
**PARENTAL PARTICIPATION & CONCERNS**	Involving parents in the preparation, transfer and handover conversation Scheduling an online meeting for complex cases, involving parents, referring and receiving care professionals
	A parent or familiar care professionals accompanying the infant during the transfer
	Asking parents if they have concerns or fears regarding the transfer
	Encouraging parents to document key information they believe is important for the receiving care professionals to know to provide optimal care. This written parental handover facilitates level II care professionals in providing optimal care

## Discussion

The results of our study provide valuable insights into the perception of safety during the transfer process of infants from a NICU to a level II neonatology department from multiple perspectives. Overall, both parents and all the different care professionals involved in this study expressed *a uniformly positive perception* regarding the degree of safety throughout the transfer process. This is a significant finding as it indicates that all stakeholders have confidence in the care process and safety measures implemented during the transfer. Importantly, there were no statistically significant differences between stakeholder groups regarding the overall degree of safety, suggesting a consistent perception of safety across the board. This finding is particularly noteworthy considering previous reports that have indicated that parents often experience stress and feelings of anxiety regarding the transfer [19–32]. The positive perception of parents in our study may be attributed to various factors, such as effective communication, a sense of trust in the healthcare team, and the overall stability of the infant’s condition at the time of transfer. It could indicate that efforts to enhance safety measures and collaboration among stakeholders have been effective in mitigating parental concerns and instilling confidence in the care provided during the transfer. This unexpected finding highlights the potential impact of interventions aimed at improving the transfer experience and ensuring the well-being of infants and their families. Reflecting on these positive findings from the appreciative Safety-II perspective, we can conclude that our research provides valuable insight into the facilitators of a positively experienced transfer process. Parents witnessed and valued the knowledge, skills, and expertise of the healthcare providers, which reinforced feelings of trust and safety. When parents were actively involved in the transfer process or when care professionals took the time to pay attention to parents and their thoughts, it was associated with a high degree of perceived safety by parents. The same was true for care professionals. When care professionals proactively made the effort to show interest in the abilities and concerns of other care professionals, it enhanced the feeling of safety.

These findings underscore the value of promoting effective, and open communication channels between parents and care professionals, as well as among care professionals. The involvement of parents in decision-making and encouraging collaborative decision-making among care professionals can facilitate problem-solving and ensure a safe transfer process. Based on these findings, we are currently exploring opportunities to further involve parents in the transfer process and strengthen the dialogue among care professionals. The impact of these interventions will be evaluated in future research.

Zooming in on different *phases of the transfer process*, once again, our results indicate that all stakeholder groups reported high levels of perceived safety. Median scores consistently ranged between 8.0 and 9.0, indicating a robust perception of safety across all phases. However, there were significant differences in perceived safety during the transfer and admission phases among different stakeholders, with parents together with ambulance personnel being the most positive in their perception. Furthermore, *parents perceived a significantly higher level of safety* during admission compared to level II care professionals. There are several potential explanations for this finding.

First, parents often develop a strong bond, a feeling of gratitude, and trust with healthcare professionals, which can contribute to their positive perception of safety during the transfer process [32]. They may feel reassured by the expertise and information provided by the professionals and by the knowledge that the transfer is being conducted under their professional guidance. In contrast, care professionals may be more aware of potential risks and complexities involved. They may have a deeper understanding of the challenges, limitations, and potential complications that can arise during the transfer of these vulnerable infants. Their professional knowledge and experience may lead to a more cautious perception of safety compared to parents. Furthermore, care professionals may experience a feeling of accountability and responsibility for the care provided. They may be more attuned to potential risks and may have a greater awareness of adverse events that can occur during transfers. This heightened awareness and responsibility may influence their perception of safety, leading to a more critical assessment.

The *differences in perceptions among care professionals* may stem from various factors, including variations in experience, training, and exposure to transfer cases. It is crucial to address these differences and foster a shared understanding of the transfer process among all care professionals involved. This can be achieved through effective communication, collaboration, education, and the establishment of clear guidelines and protocols to ensure consistent and standardized care during transfers. Our finding that level II healthcare professionals receiving information were less satisfied with the completeness and accuracy of the information compared to the NICU professionals providing the information raises important considerations regarding communication and information exchange within the transfer process. Effective communication is crucial for ensuring a smooth and safe transfer of patients. In this study, it appears that there may be a discrepancy between the expectations and perspectives of healthcare professionals providing and receiving information. The providers of information may have a better understanding of the context, patient history, and specific details relevant to the transfer. They may assume that certain information is already known or overlook elements they consider less significant. On the other hand, the receiving healthcare professionals may have different expectations and requirements regarding the information they need to provide optimal care. These differing perspectives and expectations can contribute to discrepancies in perceived completeness and accuracy, potentially leading to misunderstandings, delays, and a decreased overall perception of safety. To bridge this gap, referring healthcare professionals should have an optimal understanding of the context, nuances, and specific needs of the receiving healthcare professionals. This would allow them to tailor the information to meet those needs more effectively. When written handovers and/or diagnostic images were shared with the receiving care professionals prior to the transfer, it stimulated the perception of safety. The same applied to a handover conversation in which parents, referring, and receiving care professionals participated through an online meeting. These examples of Safety-II behaviour, where care professionals learned from previous successful transfers, facilitated the receiving care professionals and parents in adequately preparing for the transition of care. These exemplary actions simultaneously addressed the healthcare needs of the patient, the expectations of the parents, and supported level II care professionals in the delivery of optimal care.

Overall, differences in perception regarding the safety of care delivered can be attributed to a combination of personal involvement/emotional attachment, professional knowledge, and differing perspectives on what constitutes safety. Different individuals may have varying thresholds for what they consider safe or unsafe and may prioritize aspects of safety differently. Understanding these variations in perception can help healthcare professionals optimise their communication and care. The differences in perception of safety between stakeholders underscore the need for effective communication and shared decision-making throughout the transfer process. Using those different perspectives to increase knowledge regarding the improvement of safety could enhance the quality of the transfer process.

We have identified several facilitators and barriers to the perception of a high degree of safety.

The most important *facilitators* were related to (1) the optimal timing of the transfer, (2) early, consistent, transparent information exchange, (3) knowing and meeting each other’s expectations and limitations, (4) parental participation and (5) attention for concerns. *Barriers* to experiencing a high level of safety mainly revolved around two themes: (1) communication (e.g., too little, too late, inconsistent) and (2) planning/organizing (e.g., incomplete preparation). Although parents perceived the transfer as safe, many parents still experience *concerns* regarding the transfer. These concerns were predominantly related to the fear of the unknown and concerns about the comfort of their infant. Interestingly, most parents felt reluctant to share their concerns with care professionals. However, actively addressing parents’ concerns by care professionals alleviated fears and uncertainties, emphasizing the importance of stimulating open communication and support. Our study demonstrates that we can increase our understanding of safety by reflecting on daily practice from the perspective of all major stakeholders, even when safety has already been achieved and perceived. This finding aligns with Safety-II principles.

### Limitations

It is possible that parents as well as care professionals rated the degree of safety of care more positively due to the survey investigating safety itself and the attention given to safety by the department. This phenomenon is known as the Hawthorne Effect, where individuals modify their behaviour or perception when they know they are being observed or studied [48]. When respondents are aware that their opinions and perceptions of safety are being assessed through a survey, it can create a sense of importance and attention to the topic of safety. This heightened awareness can lead parents and care professionals to pay closer attention to safety measures, be more cautious, and potentially have a more positive perception of safety during their care. Furthermore, the survey itself can serve as a reminder for healthcare professionals to prioritize safety and implement safety measures more diligently. The knowledge that safety is being assessed may lead to increased vigilance and efforts to enhance safety protocols and communication, which can contribute to a more positive perception of safety among patients. It is important to acknowledge that our study’s findings may be context-specific and influenced by various factors, such as the specific healthcare setting and the effectiveness of local protocols and guidelines. Our NICU transfers a high percentage of infants to level II hospitals for convalescent care. The transfer of infants from a NICU to a level II department is therefore a frequently occurring event. Nonetheless, the consistent perception of safety among stakeholders provides valuable insights into the effectiveness of current practices and highlights the potential for further improvements in the transfer process.

Healthcare professionals were asked to complete the questionnaire immediately after the transfer, while parents completed the survey one week after the transfer. Although this could have introduced a recall bias among parents, it was a deliberate design choice. Given that parents perceive the transfer of their child as highly stressful, asking them to complete a survey in an interview setting immediately afterwards would have been inappropriate.

We did not collect data on morbidity, as we found no strong empirical or theoretical link between these confounders and patient safety. The perception of safety among parents and healthcare professionals is inherently subjective and not solely determined by clinical characteristics or disease severity. Parents may interpret severity based on emotional distress and prior experiences, while healthcare providers’ perceptions may vary depending on their experience, workload, and available resources. Given these complexities and ethical constraints, we did not attempt to collect or analyse these variables.

## Conclusion

*In conclusion*, our study highlights the positive perception of safety regarding the transfer of infants from a NICU to a level II neonatology department among parents and care professionals. Despite some variations in perceptions among stakeholder groups, overall, high levels of perceived safety were observed. The differences in perception of safety between stakeholders underscore the need for effective communication, reflection and shared decision-making throughout the transfer process. Understanding the factors that influence safety perception could help healthcare professionals to optimise the transfer process and ensure the well-being of infants and their families. Further research and implementation of targeted interventions based on these findings can contribute to ongoing efforts to improve the safety and quality of care during transfers in neonatal settings.

## Electronic supplementary material

Below is the link to the electronic supplementary material.

Supplementary Material 1

Supplementary Material 2
