# Supplementary material for: The perception of safety regarding the transfer of infants from the neonatal intensive care unit to a level II neonatology department: a mixed-method cohort study using a Safety-II approach
Source: BMC Pediatr. 2025 Mar 17;25:211. doi: 10.1186/s12887-025-05537-4 (PMC11912773; doi:10.1186/s12887-025-05537-4)
Supplement: Supplementary file 1 — Supplementary Material 1 [file 12887_2025_5537_MOESM1_ESM.docx]

**ADDITIONALE FILE 1: Level of neonatal care categories.**

| **LEVEL OF CARE** | **CARE DELIVERED** | **CARE PROFESSIONALS** |
| --- | --- | --- |
| **LEVEL I**  **Well new born** neonatology department | - Provide neonatal resuscitation at every delivery - Evaluate and provide postnatal care to stable term newborn infants - Stabilize and provide care for infants born 35–37 wk gestation who remain physiologically stable - Stabilize newborn infants who are ill and those born at <35 wk gestation until transfer to a higher level of care | Pediatricians, family physicians, nurse  practitioners, and other advanced practice registered nurses |
| **LEVEL II**  **Special care** neonatology department | *Level I capabilities plus:*   - Provide care for infants born ≥32 wk gestation and weighing ≥1500^A^ g who have physiologic immaturity or who are moderately ill with problems that are expected to resolve rapidly and are not anticipated to need subspecialty services on an urgent basis - Provide care for infants convalescing after intensive care - Provide mechanical ventilation for brief duration (<24 h) or continuous positive airway pressure or both - Stabilize infants born before 32 wk gestation and weighing less than 1500^A^ g until transfer to a neonatal intensive care facility | *Level I health care professionals plus*  Pediatric hospitalists, neonatologist,  and neonatal nurse practitioners/physician assistant. |
| **Level III**  **NICU** | *Level II capabilities plus:*   - Provide sustained life support - Provide comprehensive care for infants born <32 wks   gestation and weighing <1500^A^ g and infants born at all gestational ages and birth weights with critical illness   - Provide prompt and readily available access to a full range of pediatric medical subspecialists, pediatric surgical specialists, pediatric anesthesiologists, and pediatric opthalmologists - Provide a full range of respiratory support that may include conventional and/or high-frequency ventilation and inhaled nitric oxide - Perform advanced imaging, with interpretation on an urgent basis, including computed | *Level II health care professionals plus:*  Pediatric medical subspecialists,  pediatric anesthesiologists,  pediatric surgeons, and pediatric  opthalmologists. |
| **LEVEL IV**  **Regional NICU** | *Level III capabilities plus:*  Located within an institution with the capability to provide:   - Surgical repair of complex congenital or acquired conditions - Maintain a full range of pediatric medical subspecialists,   pediatric surgical subspecialists, and pediatric anesthesiologists at the site   - Facilitate transport and provide outreach education | Level III health care professionals plus:  Pediatric surgical subspecialists |

^A:^ In the Netherlands, level II facilities provide care to infants born at ≥32 wk gestation and weighing ≥**1200** grams. Level II post intensive care/high care department provide convalescent care to infants born at ≥30 wk gestation and weighing ≥ **1000** grams. [15]

**ADDITIONAL FILE 2: SURVEYS**

| **PARENTS** | **Aspect** | **Question** | **Answer** | |
| --- | --- | --- | --- | --- |
|  | **Preparation** | The care professionals of the NICU prepared me/us sufficient and timely for the transfer | Yes / No | Additional information |
|  |  | Could you discuss concerns with the care professionals of the NICU? | Yes / No | Additional information |
|  |  | Were your concerns taken seriously and did they lead to actions to diminish those concerns? | Yes / No | Additional information |
|  |  | The receiving care professionals were well informed about my child by the NICU professionals? | Yes / No | Additional information |
|  |  | How do you rate the preparation of the transfer? | Scale 1 -10  (10 most optimal) | |
|  | **Transfer** | The transfer to the other hospital was conducted safely? | Yes / No | Additional information |
|  |  | Did you have concerns regarding the safety of the transfer prior to the transfer? | Yes / No | Additional information |
|  |  | Were you able to share those concerns with the care professionals? | Yes / No | Additional information |
|  |  | What was the cause of those concerns? | Open question | |
|  |  | How do you rate the safety of your child during the transfer? | Scale 1 -10  (10 most optimal) | |
|  |  | Was the transfer conducted at the most optimal moment? | Yes / No | Additional information |
|  |  | Why was another moment more optimal? | Open question | |
|  |  | Do you understand why your child was transferred when he/she was being transferred? | Yes / No | Additional information |
|  | **Admission level II dep.** | How do you rate the admission to the level II department? | Scale 1 -10  (10 most optimal) | |
|  |  | Remarks regarding admission level II department? | Yes / No | Additional information |
|  | **Facilitator & barriers** | Which factors contributed to the safety of the transfer of your child in a positive manner? | Open question | |
|  |  | What were points of improvement regarding the overall transfer process? | Open question | |
| **CARE PROFESSIONALS NICU** | **Aspect** | **Question** | **Answer** | |
|  | **Preparation** | The preparation of parents for the transfer by me/my colleague was sufficient and timely | Likert scale  1-5 | Additional information |
|  |  | I asked parents if they had concerns regarding the transfer and if so, I took action to diminish those concerns | Likert scale  1-5 | Additional information |
|  |  | The receiving care professionals were well informed about the patient by me/ my colleague | Likert scale  1-5 | Additional information |
|  |  | How do you rate the preparation of the transfer? | Scale 1 -10  (10 most optimal) | |
|  | **Transfer** | The transfer to the other hospital was conducted safely? | Likert scale  1-5 | Additional information |
|  |  | Did you have concerns regarding the safety of the transfer prior to the transfer? | Yes / No | Additional information |
|  |  | Were you able to share those concerns with colleagues? | Yes / No | Additional information |
|  |  | What was the cause of those concerns? | Open question | |
|  |  | How do you rate the safety of the patient during the transfer? | Scale 1 -10  (10 most optimal) | |
|  |  | Was the transfer conducted at the most optimal moment? | Likert scale  1-5 | Additional information |
|  |  | Why was another moment more optimal? | Open question | |
|  | **Facilitator & barriers** | Which factors contributed to the safety of the transfer in a positive manner? | Open question | |
|  |  | What were points of improvement regarding the overall transfer process? | Open question | |
|  | **Aspect** | **Question** | **Answer** | |
| **AMBULANCE PERSONNEL** | **Preparation** | I received sufficient information from the NICU care professionals to guarantee the safety of the patient during the transfer | Likert scale  1-5 | Additional information |
|  |  | How do you rate the preparation of the transfer? | Scale 1 -10  (10 most optimal) | |
|  | **Transfer** | A parent accompanied the patient during the transfer? | Yes / No | |
|  |  | A NICU care professional accompanied the transfer? | Yes / No | |
|  |  | The transfer to the other hospital was conducted safely? | Likert scale  1-5 | Additional information |
|  |  | Did you have concerns regarding the safety of the transfer prior to the transfer? | Yes / No | Additional information |
|  |  | Were you able to share those concerns with other care professionals? | Yes / No | Additional information |
|  |  | What was the cause of those concerns? | Open question | |
|  |  | How do you rate the safety of the patient during the transfer? | Scale 1 -10  (10 most optimal) | |
|  |  | Was the transfer conducted at the most optimal moment? | Likert scale  1-5 | Additional information |
|  |  | Why was another moment more optimal? | Open question | |
|  | **Facilitator & barriers** | Which factors contributed to the safety of the transfer of the patient in a positive manner? | Open question | |
|  |  | What were points of improvement regarding the overall transfer process? | Open question | |
| **SECRETARY PERSONNEL** | **Aspect** | **Question** | **Answer** | |
|  | **Preparation** | I received sufficient information from the care professionals to prepare the transfer safely | Likert scale  1-5 | Additional information |
|  |  | How was the communication with the ambulance control room | Likert scale  1-5 | Additional information |
|  |  | How do you rate the preparation of the transfer? | Scale 1 -10  (10 most optimal) | |
|  |  | Any remarks regarding the preparation of the transfer? | Open question | |
|  |  | Was the transfer conducted at the most optimal moment? | Likert scale  1-5 | Additional information |
|  | **Facilitator & barriers** | Which factors contributed to the safety of the transfer in a positive manner? | Open question | |
|  |  | What were points of improvement regarding the overall transfer process? | Open question | |
